# Supplementary material for: Dissociation of task engagement and arousal effects in auditory cortex and midbrain
Source: eLife. 2021 Feb 11;10:e60153. doi: 10.7554/eLife.60153 (PMC7909948; doi:10.7554/eLife.60153)
Supplement: Transparent reporting form [file elife-60153-transrepform.docx]

***eLife’s* transparent reporting form**

We encourage authors to provide detailed information *within their submission* to facilitate the interpretation and replication of experiments. Authors can upload supporting documentation to indicate the use of appropriate reporting guidelines for health-related research (see [EQUATOR Network](http://www.equator-network.org/%20)), life science research (see the [BioSharing Information Resource](https://biosharing.org/)), or the [ARRIVE guidelines](http://www.plosbiology.org/article/info:doi/10.1371/journal.pbio.1000412) for reporting work involving animal research. Where applicable, authors should refer to any relevant reporting standards documents in this form.

If you have any questions, please consult our Journal Policies and/or contact us: [editorial@elifesciences.org](mailto:editorial@elifesciences.org).

**Sample-size estimation**

- You should state whether an appropriate sample size was computed when the study was being designed
- You should state the statistical method of sample size computation and any required assumptions
- If no explicit power analysis was used, you should describe how you decided what sample (replicate) size (number) to use

Please outline where this information can be found within the submission (e.g., sections or figure legends), or explain why this information doesn’t apply to your submission:

Sample size was determined based on standards established by previous publications studying single-neuron activity during behavior, which have been adequate to demonstrate significant population effects. A traditional power analysis is not possible because noise properties of neural data are difficult to estimate a priori. Based on norms for the field, we acquired data from at least three animals and numbering at least 50 neurons per brain area. This information is provided in the analysis section of Materials & Methods.

**Replicates**

- You should report how often each experiment was performed
- You should include a definition of biological versus technical replication
- The data obtained should be provided and sufficient information should be provided to indicate the number of independent biological and/or technical replicates
- If you encountered any outliers, you should describe how these were handled
- Criteria for exclusion/inclusion of data should be clearly stated
- High-throughput sequence data should be uploaded before submission, with a private link for reviewers provided (these are available from both GEO and ArrayExpress)

Please outline where this information can be found within the submission (e.g., sections or figure legends), or explain why this information doesn’t apply to your submission:

We state sample size in the Results each time a new group of neurons is introduced, as well as in the Materials & Methods, when we describe the experimental approach. Analyses focus on a single neuron or a single neuron in a unique behavioral condition; thus these constitute biological replicates. A single outlier is indicated with an arrow in Figure 6. Neural data were included if they passed the screen for adequate isolation, as described in the Materials & Methods.

**Statistical reporting**

- Statistical analysis methods should be described and justified
- Raw data should be presented in figures whenever informative to do so (typically when N per group is less than 10)
- For each experiment, you should identify the statistical tests used, exact values of N, definitions of center, methods of multiple test correction, and dispersion and precision measures (e.g., mean, median, SD, SEM, confidence intervals; and, for the major substantive results, a measure of effect size (e.g., Pearson's r, Cohen's d)
- Report exact p-values wherever possible alongside the summary statistics and 95% confidence intervals. These should be reported for all key questions and not only when the p-value is less than 0.05.

Please outline where this information can be found within the submission (e.g., sections or figure legends), or explain why this information doesn’t apply to your submission:

The relevant statistical test is named each time significance is assessed, in the text and figure legends. P values for analysis of group data are reported to three significant digits. In some cases, neurons were classified according to whether a test was significant or not. In this case, the threshold for grouping is indicated (e.g., “p<0.05”).

(For large datasets, or papers with a very large number of statistical tests, you may upload a single table file with tests, Ns, etc., with reference to sections in the manuscript.)

**Group allocation**

- Indicate how samples were allocated into experimental groups (in the case of clinical studies, please specify allocation to treatment method); if randomization was used, please also state if restricted randomization was applied
- Indicate if masking was used during group allocation, data collection and/or data analysis

Please outline where this information can be found within the submission (e.g., sections or figure legends), or explain why this information doesn’t apply to your submission:

Groups were defined by brain area of recording and behavior condition (i.e., the task being performed during neural recording). Random grouping was only used in permutation tests that required random shuffling of the variable of interest. In this case, group identity was random but the size of each group was kept the same. Masking was not used.

**Additional data files (“source data”)**

- We encourage you to upload relevant additional data files, such as numerical data that are represented as a graph in a figure, or as a summary table
- Where provided, these should be in the most useful format, and they can be uploaded as “Source data” files linked to a main figure or table
- Include model definition files including the full list of parameters used
- Include code used for data analysis (e.g., R, MatLab)
- Avoid stating that data files are “available upon request”

Please indicate the figures or tables for which source data files have been provided:

The full dataset will be shared in a public repository (Zenodo) as part of the final publication. Code used for analysis is provided in a git repository. Source data and scripts for specific figures will be included with the final published data.
